# Holocaust Handed Down: Investigating the Relationship Between Inherited Trauma and Alzheimer's Disease Incidence Through Family Functioning

**DOI:** 10.1002/alz70860_103127

**Published:** 2025-12-23

**Authors:** Rachel L Geisel

**Affiliations:** ^1^ Columbia University Mailman School of Public Health, New York, NY, USA

## Abstract

**Background:**

11β‐hydroxysteroid dehydrogenase type 2 (11β‐HSD2) may be affected by malnutrition or extreme stress. A previous study observed lower levels of 11β‐HSD2 in Holocaust survivors compared to age‐matched Jewish controls. When individuals experience adversity at a young age, their 11β‐HSD2 levels do not return to normal post‐trauma. High levels of 11β‐HSD2 can increase intracellular glucocorticoid levels and have been associated with ADRD in animal models. During pregnancy, 11β‐HSD2 protects the fetus from circulating maternal cortisol in the placenta. The high levels of 11β‐HSD2 in offspring may represent an adaptation to protect the fetus from low levels of 11β‐HSD2 in their mothers. In a cohort study of offspring of Holocaust survivors, the highest levels of 11β‐HSD2 were associated with maternal exposure to the Holocaust. It is feasible that both biological and psychoemotional pathways are acting in concert in the children of survivors of significant traumatic events that increase risk for ADRD later in the life course.

**Method:**

Using longitudinal data from Washington Heights Inwood Community Aging Project, we will examine brain integrity indicators via MRI data. MRI will provide information on ICV and total weighted hippocampal volume through T1‐weighted images. A cortical thickness composite will be created using FreeSurfer T1‐weighted images. Total white matter hyperintensity volumes will be measured through T2‐weighted FLAIR images. Additional children of Holocaust survivors will be recruited to assess language ability through confrontation naming, letter and category fluency, verbal abstract reasoning, repetition, and comprehension. These additional participants will be administered a Family Resilience Assessment Scale Test and additional questions related to family functioning.

**Result:**

The primary aim of this study is to estimate the association between intergenerational trauma via FRAS score and brain integrity indicators. We except negative FRAS scores will be associated with brain integrity indicators consistent with ADRD.

**Conclusion:**

Elucidating the pathway between intergenerational trauma and ARRD incidence will provide new and important insight into the aging process and risk factors for ADRD.

